# Isolation of a *Lactobacillus paracasei* Strain with Probiotic Attributes from Kefir Grains

**DOI:** 10.3390/biomedicines8120594

**Published:** 2020-12-11

**Authors:** Stavros Plessas, Despoina Eugenia Kiousi, Marina Rathosi, Athanasios Alexopoulos, Yiannis Kourkoutas, Ioanna Mantzourani, Alex Galanis, Eugenia Bezirtzoglou

**Affiliations:** 1 Laboratory of Food Processing, Faculty of Agricultural Development, Democritus University of Thrace, 68200 Orestiada, Greece; alexopo@agro.duth.gr (A.A.); imantzou@agro.duth.gr (I.M.); 2 Department of Molecular Biology and Genetics, Faculty of Health Sciences, Democritus University of Thrace, 68100 Alexandroupolis, Greece; dkiousi@mbg.duth.gr (D.E.K.); rathosim@gmail.com (M.R.); agalanis@mbg.duth.gr (A.G.); 3 Laboratory of Applied Microbiology and Biotechnology, Department of Molecular Biology and Genetics, Democritus University of Thrace, 68100 Alexandroupolis, Greece; ikourkou@mbg.duth.gr; 4 Laboratory of Hygiene and Environmental Protection, Medical School, Faculty of Health Sciences, Democritus University of Thrace, 68100 Alexandroupolis, Greece; empezirt@med.duth.gr

**Keywords:** kefir, lactic acid bacteria, probiotics, adherence, cancer cells, apoptosis, flow cytometry

## Abstract

Κefir is a rich source of potentially probiotic bacteria. In the present study, firstly, in vitro screening for probiotic characteristics of ten lactic acid bacteria (LAB) isolated from kefir grains was performed. Strain AGR 4 was selected for further studies. Molecular characterization of strain AGR 4, confirmed that AGR 4 belongs to the *Lactobacillus paracasei* (reclassified to *Lacticaseibacillus paracasei* subsp. *paracasei*) species. Further testing revealed that *L. paracasei* AGR 4 displayed adhesion capacity on human adenocarcinoma cells, HT-29, similar to that of the reference strain, *L. casei* ATCC 393. In addition, the novel strain exerted significant time- and dose-dependent antiproliferative activity against HT-29 cells and human melanoma cell line, A375, as demonstrated by the sulforhodamine B cytotoxicity assay. Flow cytometry analysis was employed to investigate the mechanism of cellular death; however, it was found that AGR 4 did not act by inducing cell cycle arrest and/or apoptotic cell death. Taken together, these findings promote the probiotic character of the newly isolated strain *L. paracasei* AGR 4, while further studies are needed for the detailed description of its biological properties.

## 1. Introduction

Probiotics are defined as viable microorganisms that can confer health benefits to the host, when consumed in appropriate amounts [1]. The consumption of probiotics has been linked to the alleviation of gastrointestinal conditions, such as Crohn’s disease, ulcerative colitis, irritable bowel syndrome, as well as antibiotic-associated diarrhea in children and adults [2]. Additionally, accumulating evidence suggests that probiotics may exert their effects on extraintestinal sites as well [3]. The efficacy of probiotic supplementation depends on strain- and host-specific factors. Recent studies have highlighted these aspects and introduced the need for a case-by-case approach for the study of the molecular and cellular events that are responsible for the effects induced by each individual strain [4]. The key functions of probiotics include antimicrobial and immunomodulatory activity, modulation of gut microbiota function and structure, and protection of the intestinal barrier integrity [5]. Cellular attachment seems to be an important feature for the mediation of probiotic action. It is predominantly involved in the competitive exclusion of enteropathogens, as observed in vitro and in vivo [6,7], while it seems to enhance the antiproliferative potential of certain probiotic strains against cancer cells [8]. However, recent data suggests that colonization is not a prerequisite for the implementation of probiotic actions, as transient adherence to the gut epithelium can be sufficient [9]. The antiproliferative and cytotoxic events induced by certain strains are frequently attributed to the induction of cell cycle arrest and apoptotic cell death [10]. Probiotics can induce these effects by interfering with cellular cascades that are involved in cell cycle progression and proliferation, as shown in a plethora of in vitro mechanistic studies [10]. However, specific strains can also be responsible for the activation of other regulated cell death pathways, such as autophagy-induced [11] or immunogenic cell death [12].

Potentially probiotic strains can be isolated from the human gastrointestinal tract (GIT), breast milk or feces [13]. In this vein, the human gastric isolate *L. rhamnosus* UCO-25A demonstrated immunomodulatory and anti-*Helicobacter* properties [14], whereas, *L. fermentum* CECT5716, isolated from breast milk was shown to promote growth in animal models of chronic malnutrition [15]. Novel bacterial strains with potential probiotic attributes can also be derived from fermented dairy and non-dairy products, including meat, fruits, cheese and fermented milks, such as kefir [13]. Kefir is an acidic, self-carbonated, low-alcoholic drink, made from the fermentation of milk with kefir grains. The consumption of kefir drink has been linked to several health benefits (improved digestion, antihypertensive and hypocholesterolemic activity), and for that reason, many efforts have been focused on the isolation and characterization of the kefir microflora [16]. Indeed, some of the strains hosted in this matrix exhibit antimicrobial, anti-inflammatory, antioxidant, and immunomodulatory activities [16,17]. These effector strains are usually lactic acid bacteria (LAB), and various yeasts [18,19].

The aims of our study were—firstly, isolation of novel LAB strains from kefir grains and in vitro screening for probiotic characteristics; secondly, molecular genotyping of the strain with the best probiotic scores; and finally, investigation of its cellular mechanisms of action, including its adhesion properties and antiproliferative effects against two human cancer cell lines, and the potential molecular pathways involved.

## 2. Materials and Methods

### 2.1. Kefir Grains Isolation

Kefir grains were sourced from a commercially available kefir beverage. Firstly, separation of the grains from the drink was performed by straining and then the grains were washed once with sterile de Man, Rogosa, and Sharp (MRS) broth (Sigma-Aldrich, Taufkirchen, Germany).

### 2.2. Isolation of LAB Strains

Homogenization of the kefir grains was performed with 250 mL (0.1% *w*/*v*) peptone water in filtered stomacher bags for 3 min. Afterwards, the contents were serially diluted and plated into MRS agar (Sigma-Aldrich) and incubated at 37 °C for 48 h. Individual colonies were picked and cultivated in MRS broth (Sigma-Aldrich) at 37 °C for 48 h. The isolates were further purified by streak plating. Morphological and staining characteristics were used for the preliminary identification of strains, as previously described [20].

### 2.3. Bacterial Strains and Culture Conditions

*L. paracasei* K5 was isolated from Greek feta-type cheese [20], whereas strains *L. paracasei* SP3 [21], SP5 [22] and AGR 4 (this study), were isolated from kefir grains. The reference strains *L. plantarum* ATCC 14,917 and *L. casei* ATCC 393 were purchased from ATCC (LGC Standards, Middlesex, UK). All strains were grown anaerobically in MRS broth (Sigma-Aldrich) at 37 °C.

### 2.4. Resistance to Low pH, Pepsin, Pancreatin and Tolerance to Bile Salts

To evaluate resistance to pH, bacterial cells were grown overnight (18 h), collected by centrifugation at 10,000× *g*, 4 °C for 5 min, the pellets were washed twice with phosphate-buffered saline (PBS) buffer (Biosera, Boussens, France) (pH 7.2), resuspended in PBS adjusted to pH 2.0, 3.0 or 4.0 and incubated, anaerobically, for 0 to 2 h at 37 °C. Similarly, for the evaluation of resistance to pepsin and pancreatin the pellets were resuspended in PBS with the addition of pepsin (3 mg/mL; Sigma-Aldrich), or pancreatin USP (1 mg/mL; Sigma-Aldrich) at 37 °C for 0 to 3 h [23]. Finally, tolerance to bile salts was measured following the cultivation of the strains in PBS solution (pH 8.0) with 0.5% (*w*/*v*) bile salts extracts (Oxoid LP0055; Thermo Fisher Scientific, Waltham, MA, USA), consisting mainly of sodium glycocholate and sodium taurocholate. Viable colony count was used as a measure of bacterial resistance to the conditions tested.

### 2.5. Antibiotic Susceptibility

Antibiotic susceptibility expressed as minimum inhibitory concentration (MIC) was assessed using the M.I.C. Evaluator^®^ strips [20]. Tests included the following antibiotics: amoxycillin (256–0.015 μg/mL), amoxycillin + clavulanic acid (256–0.015 μg/mL), ampicillin (256–0.015 μg/mL), clindamycin (256–0.015 μg/mL), erythromycin (256–0.015 μg/mL), gentamycin (1024–0.06 μg/mL), metronidazole (256–0.015 μg/mL), tetracycline (256–0.015 μg/mL), tigecycline (256–0.015 μg/mL) and vancomycin (256–0.015 μg/mL). Plates with Mueller–Hinton agar (Sigma-Aldrich) were inoculated with a bacterial suspension of 1.5 × 10^8^ CFU/mL, then the strips were applied, and the plates were incubated at 37 °C for 24 h. The mean ± standard deviation of the MIC was recorded according to the manufacturer instructions.

### 2.6. Molecular Characterisation

Molecular characterization of the selected LAB strain was performed as previously described [20]. In brief, DNA isolation was performed using a DNA isolation kit (Macherey Nagel, Düren, Germany). PCR assay was carried out with primers P1 and P2 of the V1–V3 hypervariable region of *16S rRNA* gene, as described before [24]. Purification of the PCR products was performed with a PCR extraction kit (Macherey-Nagel). Then, the purified PCR products were sequenced (VBC-Biotech, Austria) and analyzed using the BLAST software in the GenBank database. For the differentiation of *L. paracasei* and *L. casei* species, a multiplex PCR assay targeting the *tuf* gene was employed, using primers PAR, CAS, RHA and CPR [25].

### 2.7. Human Cancer Cell Lines

Human colorectal adenocarcinoma cell line HT-29 was purchased from ATCC. Cells were cultured in Roswell Park Memorial Institute (RPMI)-1640 medium enriched with GlutaMAX™, 10% fetal bovine serum (FBS), 100 μg/mL streptomycin and 100 U/mL penicillin (all from Thermo Fisher Scientific). Human melanoma cell line, A375, was purchased from ATCC and cultured in high glucose Dulbecco’s Modified Eagle Medium (DMEM) supplemented with 10% FBS, 100 μg/mL streptomycin and 100 U/mL penicillin (all from Thermo Fisher Scientific). Both cell lines were incubated in a humidified atmosphere at 37 °C, 5% CO_2_ under sterile conditions.

### 2.8. Quantitative Adhesion Assay

The quantitative adhesion assay was performed as described previously, with minor modifications [26]. HT-29 cells were seeded in 24-well plates at a density of 35 × 10^4^ cells per well and incubated for 14 days to form a monolayer. A day prior to the treatments, the cell monolayer was washed with PBS and fresh medium without antibiotics was added. The next day, 10^7^ CFU/mL of viable *Lactobacillus* AGR 4 or *L. casei* ATCC 393 cells were added to each well, with each strain being tested in duplicate. After 4 h of co-incubation at 37 °C, the cells were washed with PBS and lysed with 1% Triton X-100 (Sigma-Aldrich). The lysates were serially diluted in Ringer’s solution (Lab M, Lancashire, UK), plated on MRS agar, and incubated at 37 °C for 72 h. For the calculation of adhesion values the following formula was applied: % Adhesion = (V_B_/V_A_) × 100, where V_A_ is the initial viable count of bacteria tested (10^7^ CFU/mL), and V_B_ is the viable bacteria count, obtained from the HT-29 cells. Colony forming units per milliliter (CFU/mL) was used as viable count measure that was determined with the formula: CFU/mL = (no. of colonies × dilution factor)/volume of culture plate.

### 2.9. Cell Proliferation Assay

The sulforhodamine B (SRB) cytotoxicity assay was employed to investigate the antiproliferative potential of *Lactobacillus* AGR 4 against HT-29 and A375 cells. Cells were seeded in 96-well plates at a density of 7000 cells per well and were incubated overnight. The next day, the wells were washed with PBS and the bacteria were added in the wells at a density of 10^6^ or 10^7^ CFU/mL. After 24 or 48 h incubation the cells were washed with PBS, fixed by 50% (*w*/*v*) cold trichloroacetic acid (TCA) (Fluka, Buchs, Switzerland) and stained with 0.4% (*w*/*v*) SRB (Sigma-Aldrich) diluted in 1% (*v*/*v*) acetic acid (Scharlau, Barcelona, Spain). Removal of the excessive dye was achieved by washing with 1% (*v*/*v*) acetic acid. The bound dye was dissolved in 10 mM Tris base (Sigma-Aldrich). For the measurement of the absorbance at 570 nm a multiplate reader was used (Tecan, Männedorf, Switzerland). For the calculation of the cellular survival the following formula: ((sample OD_570_-media blank OD_570_)/(mean control OD_570_-media blank OD_570_)) × 100 was applied.

### 2.10. Evaluation of Pro-Apoptotic Activity by Flow Cytometry

Analysis of apoptosis in HT-29 cells treated with the probiotic cells or fresh medium (control group) was performed by flow cytometry after annexin V and propidium iodide staining as described before [27]. Briefly, HT-29 cells were seeded at a density of 10^6^ cells per 100 mm plate and were allowed to attach to the surface of the plate overnight. Then, the cells were washed with PBS and treated with 10^7^ CFU/mL of *Lactobacillus* AGR 4 or 10^7^ CFU/mL of *L. casei* ATCC 393 for 48 h. Two days later, the cells were collected, washed with PBS and stained with propidium iodide and annexin V-FITC (Trevigen, Gaithersburg, MD, USA) according to manufacturer’s instructions (Thermo Fisher Scientific). Flow cytometry analysis was performed twice. Data analysis was performed with FlowJo V10 software (BD Biosciences, San Jose, CA, USA). The percentage of apoptotic cells was calculated using the formula: (Ann V-FITC^+^ cells/Total no of cells) × 100.

### 2.11. Assessment of Cell Cycle Progression by Flow Cytometry

HT-29 cells were seeded at a density of 10^6^ cells in 100 mm culture plates, and cultured for 24 h in cell culture medium, followed by a 24-h serum starvation period. Afterwards, the cells were washed with PBS and treated with 10^7^ CFU/mL of *Lactobacillus* AGR 4 or *L. casei* ATCC 393 for 48 h. The cells were collected with trypsinization, centrifuged at 600× *g* for 5 min and washed with PBS. Pellets were resuspended in 1 mL of ice-cold 75% (*v*/*v*) ethanol and were left overnight at –20 °C. Then, the cells were centrifuged, washed with PBS, counted using a hemocytometer, and 10^6^ cells/mL were diluted in 50 μL of 100 μg/mL RNAse A (Sigma-Aldrich). Finally, 400 μL of 50 μg/mL propidium iodide (Sigma-Aldrich) were added in each sample and the cells were incubated in the dark, at room temperature for 40 min. DNA content and cell-cycle phases were analysed by flow cytometry (Thermo Fisher Scientific). Further analysis was performed with FlowJo V10 software (BD Biosciences, San Jose, CA, USA).

### 2.12. Statistical Analysis

Experiments were repeated three times unless otherwise specified, and the results are expressed as the average ± SD. Comparisons of viabilities or antibiotic susceptibilities among the various strains were performed using the analysis of variance (ANOVA) procedure with post hoc comparisons (Tukey’s HSD). Statistical differences from the quantitative adhesion assay, cytotoxicity assay and flow cytometry were analyzed using Student’s t-test. A *p*-value <0.05 was considered statistically significant. All statistical analyses were carried out using SPSS v20 (IBM Corp., Armonk, NY, USA).

## 3. Results and Discussion

### 3.1. Isolation of LAB Strains from Kefir Grains and In Vitro Screening for Probiotic Properties

**Figure 1:**
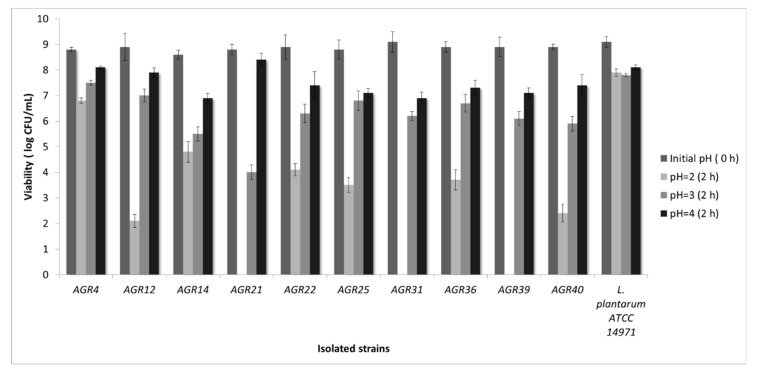
Evaluation of strain viability after exposure to low pH for 0 to 2 h.

Kefir grains represent a complex culture that has been used in various fermentation processes [20,28]. Kefir grains are rich in potentially probiotics microorganisms [29], and are shown to exhibit significant health-promoting effects [16,17]. In the present study, 10 LAB strains were isolated from kefir grains and in vitro screening for probiotic properties was performed. Firstly, evaluation of their resistance to low pH was performed. *L. plantarum* 14,917 served as a reference strain [20] (Figure 1). At pH 4, all strains had high survival rates. Nevertheless, a significant reduction of cell viability was noted in most tested LAB strains at pH 2. However, AGR 4 maintained high survival rate in high levels (6.8 log CFU/mL). This is an important finding as a probiotic strain should persist at pH 3.0 or even lower [20].

**Figure 2:**
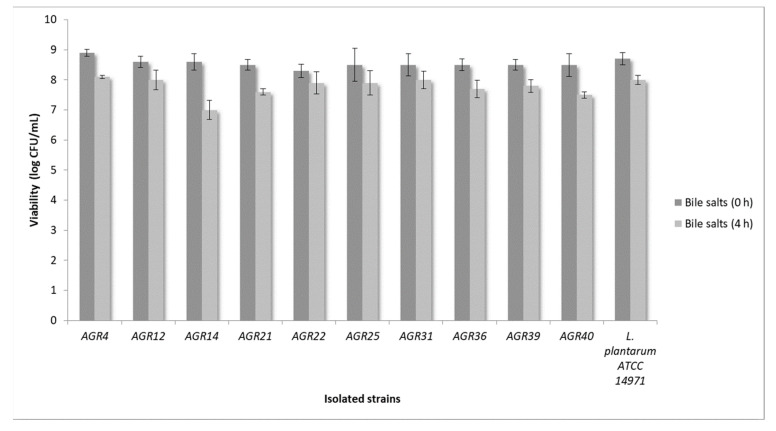
Assessment of strain viability after exposure to bile salts for 0 to 4 h.

**Figure 3:**
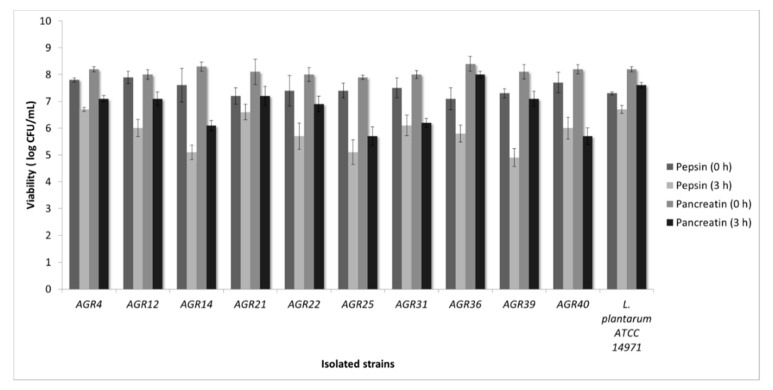
Assessment of strain viability after exposure to pepsin and pancreatin for 0 to 3 h.

Next, we tested tolerance of the isolated strains to pepsin, pancreatin and bile salts. Regarding tolerance to pancreatin, all strains retained their viability at high levels (Figure 2). Moreover, resistance to bile salts after 4 h of exposure, was recorded for all tested strains (Figure 3). Accordingly, previous studies showed that *L. paracasei* strains K5 and SP5, also isolated from kefir grains, exhibited increased resistance against pepsin and pancreatin, comparable to that of the reference strain *L. plantarum* 14,971 [20,22]. Pepsin, pancreatin and bile salts play a fundamental role in food digestion. More specifically, pepsin is an enzyme that catalyzes protein digestion. Its production is stimulated by gastric acid. Pancreatin is produced by the exocrine cells of the pancreas and plays an important role in lipid metabolism, while bile salts contribute to the digestion and absorption of fats. In that sense, tolerance to these enzymes, as well as resistance to bile salts is a prerequisite for probiotic efficacy [26]. However, the ability of potentially probiotic strains to withstand these harsh conditions should also be tested in live organisms. In this context, previous in vivo studies have demonstrated the survival of *L. casei* ATCC 393 during gastrointestinal transit in Wistar rat mice [9].

### 3.2. Safety Profile—Antibiotic Susceptibility

Antibiotic susceptibility of the isolated LAB against 10 common antibiotics was tested, as previously described [20]. The objective was a rough safety assessment of the strains based on their resistant phenotypes since there is not available a widely acceptable methodology, cut-offs or breakpoints, besides those published by the European Food Safety Authority (EFSA) [30]. The minimum inhibitory concentration for each one of the ten strains and antibiotics was estimated based on the diffusion in Mueller–Hinton agar plates, and their mean values ± standard deviation is presented in Table 1. Almost all strains exhibited the same resistant phenotype or a marginally lower than the one recorded for *L. plantarum* 14,917. An inherent resistance to vancomycin and metronidazole similar to that observed in the present study has been reported before [20,31]. Moreover, in our study shared resistance to tetracycline was recorded for all tested strains, in agreement with other similar studies [32,33]. It should be noted, however, that bacterial strains carrying intrinsic resistance (per se) present a minimal risk for horizontal spread of the resistant genes, and thus may be used as a feed additive [30]. 

### 3.3. Molecular Characterization of Lactobacillus Strain AGR 4

The *Lactobacillus* strain AGR demonstrated the best performance in the in vitro screening and was selected for further studies. Firstly, molecular characterization was performed. A variable region of the 16S rRNA gene was amplified, sequenced, and analyzed using the BLAST (Basic Local Alignment Search Tool) software. Bioinformatic analysis showed that AGR 4 exhibited 99% similarity to *L. casei* and *L. paracasei* species (data not shown). Then, multiplex PCR with primers PAR, CAS, RHA and CPR revealed that strain AGR 4 exhibited the characteristic pattern of *L. paracasei* species, with two unique amplicons at 240 bp and 520 bp, respectively (Figure S1). Thus, strain AGR 4 was identified as a *L. paracasei* species member and was named *L. paracasei* AGR 4. It should be noted that the nomenclature and taxonomy of many *Lactobacillus* species has been revisited, recently, owing to the rapid advancements of genomic and metagenomic technologies and their application in probiotic research. In this case, *L. paracasei* have been reclassified to *Lacticaseibacillus paracasei* subsp. *paracasei* [34].

### 3.4. Αdhesion of L. paracasei AGR 4 on Epithelial Colon Cancer Cells

One of the criteria for probiotic action is adherence to the gastrointestinal mucosa or epithelium. The attachment capacity of probiotic strains can be determined in both in vitro and in vivo experimental setups. Differentiated epithelial colon cancer cells are usually preferred for preliminary experiments due to their simplistic nature [35]. To this aim, the adhesion capacity of *L. paracasei* AGR 4, was tested on differentiated HT-29 monolayers. The quantitative adhesion assay revealed that 4 h treatments with *L. paracasei* AGR 4 at a density of 10^7^ CFU/mL resulted to similar adhesion levels to that of the reference strain *L. casei* ATCC 393 (Table 2). Notably, adhesion of *L. casei* ATCC 393 to epithelial colon cells has been confirmed previously by both confocal [27] and scanning electron microscopy [36]. It is worth mentioning that probiotic attachment on cancer cell lines is highly variable. For example, *Pediococcus pentosaceus* SC28 and *L. rhamnosus* GG exhibited adhesion rates of 4.45% and 6.30%, respectively, on HT-29 cells while *L. brevis* KU15151 had a slightly higher attachment rate of 6.87% [37]. Another study showed that the attachment ability of different *P. pentosaceus* strains on adenocarcinoma cancer cells Caco-2, was strain-specific and spanned from 0 to 16% [38]. Additionally, animal and human studies have showed that probiotics present a site-specific colonization pattern. More specifically, oral administration of immobilized or free *L. casei* ATCC 393 resulted in the specific colonization of caecum and colon of Wistar rats, as revealed by multiplex PCR assay [9]. In this context, qPCR-based enumeration of probiotics in tissues of C57BL/6 mice after treatments with the poly-biotic Supherb Bio-25, which is consisted of *L. casei* subsp. *paracasei, L. plantarum, L. rhamnosus, L. lactis, L. casei* subsp. *casei, S. thermophilus, B. breve, B. longum* subsp. *longum, B. bifidum,* and *B. longum* subsp. *infantis*, showed a similar colonization preference for the lower gastrointestinal tract. Indeed, the strains more readily adhered to the cecum, the proximal and distal colon. These results were replicated in a human study, where healthy individuals consumed the same probiotic mix. Interestingly, an interindividual difference in colonization patterns was also observed and was attributed to several microbiota and host genetics factors [4]. It is important to mention, however, that transient adherence to the gut is sufficient for probiotics to exert their beneficial effects. Indeed, the volunteers that consumed Supherb Bio-25 had altered transcriptional profiles in the gut [39].

### 3.5. L. paracasei AGR 4 Induces Cytotoxic Effects against HT-29 and A375 Cancer Cells

**Figure 4:**
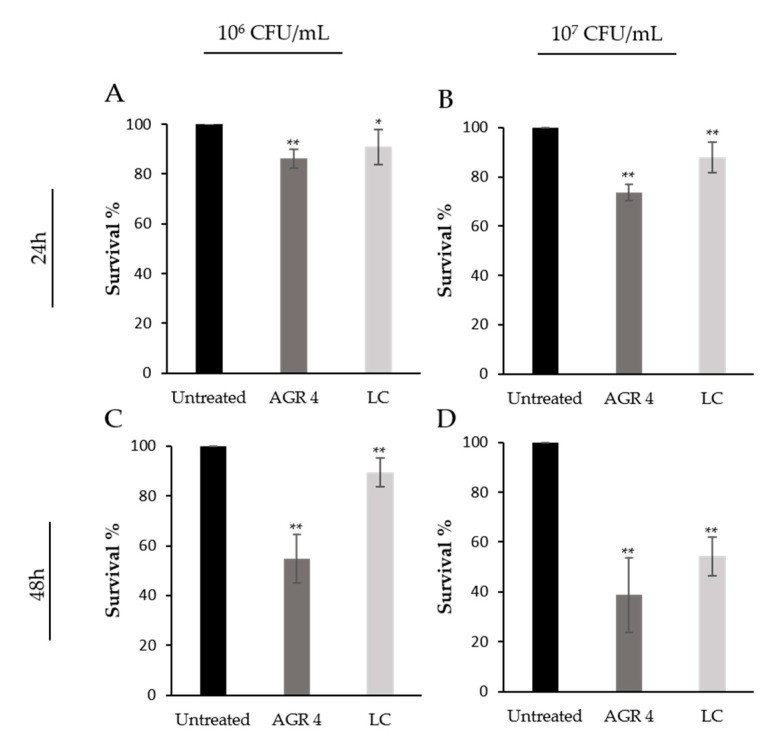
Time- and dose-dependent antiproliferative effects of viable *Lactobacillus* AGR 4 against the human adenocarcinoma cell line, HT-29, as determined by SRB assay. HT-29 cells were incubated with 10^6^ CFU/mL (**A**,**C**) or 10^7^ CFU/mL (**B**,**D**) of *Lactobacillus* AGR 4 for 24 (**A**,**B**) and 48 h (**C**,**D**). *Lactobacillus casei* (LC) ATCC 393 was included in the study as a reference strain. Data is presented as the mean ± standard deviation of three independent experiments. * *p* < 0.05; ** *p <* 0.01 compared to untreated cells.

A growing body of evidence shows that probiotics can induce antiproliferative and cytotoxic effects on cancer cells, in vitro. In this study, the cytotoxic potential of *L. paracasei* AGR 4 against HT-29 and A375 cells was assessed by the SRB colorimetric assay after treatments with two different bacterial counts (10^6^ and 10^7^ CFU/mL) in two time points (24 and 48 h). *L. casei* ATCC 393 was used as a reference strain, due to its well-characterized antiproliferative properties [40]. As shown in Figure 4, *L. paracasei* AGR 4 exerted significant reduction on HT-29 survival in a dose- and time- dependent manner. More specifically, the reduction in cell viability, recorded after 48 h incubation with 10^7^ CFU/mL of bacterial cells, reached 60% (*p* < 0.01). Accordingly, the reference strain *L. casei* ATCC 393 limited cell survival by 50%. Similar effects were, also, noted in previous studies from our lab, that investigated the antiproliferative potential of several novel probiotic strains against the human colon adenocarcinoma cell line, Caco-2. Indeed, treatments with *L. pentosus* B281, and *L. plantarum* Β282, induced a time-, dose- and strain-specific pattern of cell growth inhibition [41]. The investigation of the antiproliferative activities of probiotic strains could potentially contribute to the discovery of novel antitumor compounds to be further tested in animal models. In this line, the preliminary cytotoxic effects of viable *L. casei* ATCC 393 at concentrations of 10^8^ or 10^9^ CFU/mL against HT-29 and CT-26 (a Mus musculus colon carcinoma cell line) [40] were also demonstrated in vivo. In greater detail, BALB/c mice carrying a syngeneic subcutaneous CT26 tumor that were administered with 10^9^ CFU/mL of *L. casei* exhibited a reduction of tumor weight and volume that was accompanied by the induction of pro-inflammatory and pro-apoptotic events, in situ [42]. The translation of these findings to the clinic for the treatment of malignancies remains a challenge, however specific probiotic strains show beneficial effect by preventing perioperative infections [43] or by alleviating the gastrointestinal symptoms caused by chemotherapeutic drugs in colon cancer patients [44].

**Figure 5:**
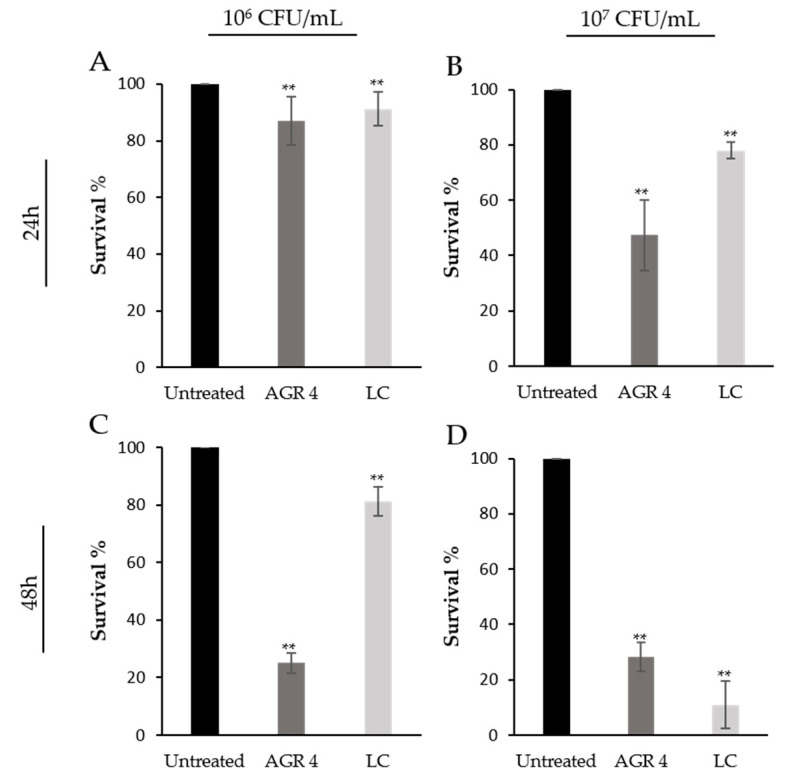
Time- and dose-dependent antiproliferative effects of viable *Lactobacillus* AGR 4 on A375 cells, measured by SRB assay. The human melanoma cell line, A375, was incubated with 10^6^ CFU/mL (**A**,**C**) or 10^7^ CFU/mL (**B**,**D**) of *Lactobacillus* AGR 4 for 24 (**A**,**B**) and 48 h (**C**,**D**). *Lactobacillus casei* (LC) ATCC 393 served as a reference strain. Data is presented as the mean ± standard deviation of three independent experiments. ** *p <* 0.01 compared to untreated cells.

The study of the beneficial effects of probiotics in skin health has gained a lot of attention in recent years. The skin hosts trillions of microorganisms which can influence its homeostasis, in combination with other environmental and genetic factors [45]. Probiotics have been shown to exert protective effects against skin carcinogenesis, by reversing ultra-violet radiation induced damage [46]. In the present study, we investigated the direct antiproliferative activity of *L. paracasei* AGR 4 against the human melanoma cell line, A375, employing the SRB assay. It was found that 48 h treatments with *L. paracasei* AGR 4 reduced cell viability to 30%, in a significant manner, as shown in Figure 5. In the same conditions *L. casei* ATCC 393 limited cell survival by 85% (*p* < 0.01) (Figure 5). To the best of our knowledge, the present study is the first to describe direct antiproliferative effects of a viable probiotic strain against A375 cells. A recent study evaluated the cytotoxic effects of *L. plantarum* L-14 extracts on this cell line. In greater detail, the authors provided evidence for the dose- and time-dependent decrease in cell viability and migration. These effects were attributed to the induction of apoptosis and downregulation of the expression of migration-related genes. Concomitantly, topical injection of the extract managed to decrease tumor weight and volume in immunodeficient BALB/c mice injected with A375 cells [47]. Today, selected probiotics strains have been added to anticancer regimes with the aim to abrogate side effects of melanoma immunotherapy [48] or to enhance the success of therapy to non-responders [49]. However, this niche of probiotic research is at its infancy and further studies are required to determine the most effective strains, doses, and routes of administration.

### 3.6. Lactobacillus Paracasei AGR 4 Does Not Induce Apoptotic Cells Death or Cell Cycle Arrest on HT-29 Cells

**Figure 6:**
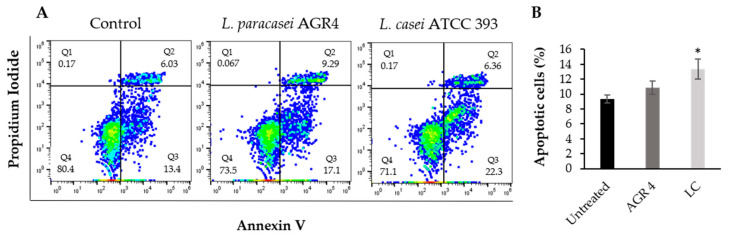
Assessment of the pro-apoptotic potential of *L. paracasei* AGR 4 via flow cytometry. HT-29 cells were co-incubated with 10^7^ CFU/mL of *Lactobacillus* AGR 4 or *L. casei* ATCC 393 (LC) for 48 h. Untreated cells incubated with culture media were used as control. (**A**) Representative flow cytometry scatter plots. Q4: live cells; Q3: early apoptotic cells; Q2: late apoptotic cells; Q1: dead cells and debris. The percentage of cells in each quadrant is also shown. (**B**) Quantitative analysis of two independent experiments. * *p* < 0.05 compared to the untreated cells.

**Figure 7:**
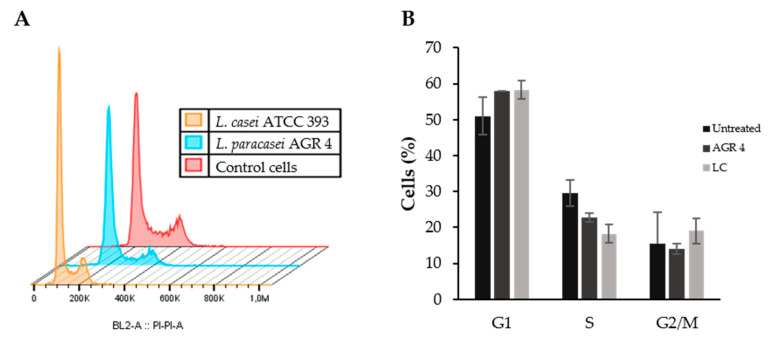
Assessment of the effect of *L. paracasei* AGR 4 in HT-29 cell cycle progression via flow cytometry. HT-29 cells were incubated with *L. paracasei* AGR 4 or *L. casei* ATCC 393 (LC) at a density of 10^7^ CFU/mL for 48 h. Cells treated with sterile culture media were used as a control. (**A**) Representative flow cytometry plot. (**B**) Quantitative analysis of two independent experiments.

The antiproliferative effects of probiotic strains are usually linked with the induction of apoptosis or/and cell cycle deregulation. For that reason, we firstly performed flow cytometry analysis on HT-29 cells treated with 10^7^ CFU/mL of *L. paracasei* AGR 4 or *L. casei* ATCC 393 for 48 h to investigate the possible induction of apoptosis. Our results show that *L. paracasei* AGR 4 did not induce apoptotic cell death (Figure 6). On the other hand, *L. casei* ATCC 393 did induce apoptotic cell death in a statistically significant manner. Similar time- and dose-dependent outcomes in the induction of such events in colon adenocarcinoma cell lines after *L. casei* treatments were also observed before [40]. Other strains that have been found to induce this mechanism of death are *L. paracasei* K5 and *L. rhamnosus* GG [27], *Escherichia coli* Nissle 1917 [50], and a probiotic cocktail consisted of *L. plantarum* (no.42), *L. reuteri* (no.100), *L. plantarum* (no.165), *L. rhamnosus* (no.195) and *L. brevis* 205 [51]. The effect is usually mediated by the probiotic-induced regulation of the expression and activity of the Bcl-2 family proteins and of major pathways involved in cell survival and proliferation, such as the Wnt/β-catenin and/or Notch signaling pathways. However, not all probiotic strains promote apoptotic cell death. For example, the strain *L. pentosus* B281, derived from olive microbiota, showed significant inhibition of the survival of Caco-2 cells that was not attributed to the induction of apoptosis [27]. However, this strain did manage to limit cell survival by inducing cell cycle arrest. For that reason, we explored the possibility of *L. paracasei* AGR 4 acting in a similar way, and thus we analyzed its effect on cell cycle progression by propidium iodide straining and DNA content measurement. This analysis did not show a significant cell cycle arrest after 48 h treatments, compared to control (untreated) cells (Figure 7). Future studies will aim at the elucidation of the exact mechanism of death induced by this novel strain. Indeed, cell death is a complex phenomenon that can be triggered by a plethora of stimuli and different cellular cascades [52]. In this vein, a surface-layer protein of *L. acidophilus* NCFM induced autophagic death by promoting the accumulation of reactive oxygen species and the modulation of the mammalian target of rapamycin and c-Jun N-terminal kinase activity in the human colon cancer cell line HCT116 [11]. Accordingly, proteomic analysis of HT-29 cells treated with *L. acidophilus* 606 cell-bound exopolysaccharides showed a significant upregulation of the expression of the proteins Beclin-1 and GRP78, which are involved in autophagic cell death [53]. Furthermore, the integral peptidoglycan (X12-PG) of *L. paracasei* subp. *paracasei* X12 could invoke important events of immunogenic cell death, namely, the translocation of calreticulin, the elevation of intracellular calcium concentrations and the release of high mobility group box 1 protein (HMGB1) [12].

## 4. Conclusions

Ten novel LAB strains were isolated from kefir grains and their probiotic properties were evaluated in a series of in vitro tests. The strain with the best attributes was molecularly assigned as *L. paracasei* AGR 4 (reclassified to *Lacticaseibacillus paracasei* subsp. *paracasei*). This strain exhibited desirable adhesion properties and potent antiproliferative activity against human colon adenocarcinoma and melanoma cell lines, which was comparable to that of the commercially available probiotic strain, *L. casei* ATCC 393. Preliminary studies on the pathways involved in the cytotoxic effects observed, did not indicate apoptotic cell death or cell cycle arrest, as a possible mechanism of action of *L. paracasei* AGR 4. Therefore, future studies should be focused on the elucidation of the mechanism of cellular death. Additionally, whole genome sequencing of the newly isolated strain would provide novel insights into its safety and efficacy profile. All in all, this strain exhibits desirable properties, and its health benefits could be additionally assessed in experimental models. Additionally, future determination of the technological properties of the strain could contribute to the development of novel dairy- or non-dairy functional products.

## Figures and Tables

**Table 1:** Assessment of antibiotic susceptibility of the isolates to common antibiotics, as determined by gradient diffusion using M.I.C. Evaluator^®^ strips. *L. plantarum* 14,917 served as a reference strain.

Agent	AGR 4	AGR 12	AGR 14	AGR 21	AGR 22	AGR 25	AGR 31	AGR 36	AGR 39	AGR 40	*L. plantarum* ATCC 14917	Cut-Off ^†^
	(Minimum Inhibitory Concentration μg/mL)											
AMX	3.48 ± 0.43 ^a^	4.20 ± 0.26 ^b^	5.05 ± 0.25 ^de^	4.98 ± 0.21 ^cde^	4.08 ± 0.31 ^b^	4.85 ± 0.29 ^de^	3.42 ± 0.21 ^a^	4.41 ± 0.55 ^bc^	4.56 ± 0.41 ^bcd^	5.11 ± 0.47 ^e^	4.55 ± 0.21 ^bcde^	n.r. ^‡^*
AMC	1.61 ± 0.31 ^de^	1.05 ± 0.3 ^ab^	1.18 ± 0.13 ^bc^	1.62 ± 0.22 ^de^	1.22 ± 0.11 ^bc^	1.65 ± 0.19 ^de^	1.38 ± 0.21 ^cd^	0.76 ± 0.07 ^a^	1.81 ± 0.09 ^ef^	0.95 ± 0.08 ^ab^	2.08 ± 0.21 ^f^	n.r. ^‡^*
AMP	1.04 ± 0.21 ^ab^	1.47 ± 0.18 ^d^	2.13 ± 0.12 ^ef^	2.38 ± 0.19 ^f^	1.13 ± 0.21 ^abc^	2.08 ± 0.11 ^e^	1.28 ± 0.09 ^bcd^	1.38 ± 0.09 ^c d^	1.52 ± 0.08 ^d^	0.95 ± 0.11 ^a^	1.21 ± 0.12 ^abc^	4 ^‡^
CLI	0.49 ± 0.05 ^a^	1.08 ± 0.14 ^bc^	0.79 ± 0.08 ^ab^	1.27 ± 0.09 ^cd^	0.89 ± 0.08 ^b^	1.13 ± 0.09 ^bc^	0.91 ± 0.11 ^b^	1.08 ± 0.19 ^bc^	0.85 ± 0.14 ^b^	1.49 ± 0.08 ^d^	0.85 ± 0.09 ^b^	1 ^‡^
ERY	0.59 ± 0.12 ^a^	1.08 ± 0.08 ^bcd^	1.06 ± 0.13 ^bc^	1.89 ± 0.07 ^e^	0.72 ± 0.09 ^a^	0.59 ± 0.07 ^a^	1.04 ± 0.08 ^bc^	1.29 ± 0.19 ^cd^	1.11 ± 0.09 ^bcd^	1.49 ± 0.29 ^de^	1.15 ± 0.21 ^cd^	1 ^‡^
GEN	4.08 ± 2.12 ^a^	4.74 ± 0.21 ^a^	5.19 ± 0.08 ^a^	5.27 ± 0.11 ^a^	4.32 ± 0.28 ^a^	4.09 ± 0.14 ^a^	4.59 ± 0.37 ^a^	5.29 ± 0.07 ^a^	4.81 ± 0.31 ^a^	4.98 ± 0.23 ^a^	4.29 ± 0.09 ^a^	32 ^‡^
MDL	189.7 ± 47.4 ^a^	148.0 ± 21.8 ^a^	195.3 ± 21.0 ^a^	201.2 ± 18.7 ^a^	195.2 ± 11.4 ^a^	200.9 ± 24.5 ^a^	195.1 ± 17.9 ^a^	198.2 ± 18.7 ^a^	200.8 ± 21.8 ^a^	199.8 ± 12.1 ^a^	199.1 ± 11.0 ^a^	n.r. ^‡^*
TET	1.24 ± 0.35 ^a^	3.41 ± 0.31 ^cd^	3.08 ± 0.28 ^bcd^	3.11 ± 0.33 ^bd^	4.08 ± 0.31 ^cde^	3.08 ± 0.43 ^bcd^	1.99 ± 0.18 ^ab^	2.48 ± 0.13 ^bc^	3.15 ± 0.23 ^cd^	4.85 ± 0.31 ^e^	4.15 ± 0.95 ^de^	4^‡^
TGC	0.48 ± 0.13 ^a^	0.58 ± 0.11 ^a^	0.52 ± 0.04 ^a^	0.52 ± 0.17 ^a^	0.58 ± 0.11 ^a^	0.53 ± 0.05 ^a^	0.57 ± 0.14 ^a^	0.61 ± 0.08 ^a^	0.42 ± 0.09 ^a^	0.65 ± 0.08 ^a^	0.71 ± 0.08 ^a^	n.r. ^‡^*
VAN	>256	>256	>256	>256	>256	>256	>256	>256	>256	>256	>256	n.r. ^‡*^

Different superscript letters in a row indicate statistically significant differences among the strains (ANOVA, Tukey’s HSD, *p* < 0.05). ^†^ Breakpoints are referred to *L. casei/paracasei* strains. ^‡^ According to EFSA, strains with a minimum inhibitory concentration (MIC) higher than the breakpoints are considered resistant (EFSA, 2012). * not required.

**Table 2:** Adhesion capacity of *Lactobacillus* AGR 4 onto HT-29 cells, assessed by quantitative analysis.

LAB Strains	Adhesion Ability (%)
*Lactobacillus* AGR 4	6.1 ± 0.14
*L. casei* ATCC 393	4.8 ± 0.85

## Supplementary Materials

The following is available online at https://www.mdpi.com/2227-9059/8/12/594/s1, Figure S1: Species-specific multiplex PCR for *Lactobacillus* AGR 4. Agarose gel electrophoresis of PCR products from multiplex PCR with primers CAS, PAR, RHA, CPR and DNA from pure cultures of *L. paracasei* K5 (line 1), *L. paracasei* SP3 (line 2), *L. paracasei* SP5 (line 3), and *Lactobacillus* AGR 4 (line 4). M: 100 bp DNA marker.
