# Supplementary figures and images for: Isolation of a Lactobacillus paracasei Strain with Probiotic Attributes from Kefir Grains
Source: Biomedicines. 2020 Dec 11;8(12):594. doi: 10.3390/biomedicines8120594 (PMC7764135; doi:10.3390/biomedicines8120594)

*L. paracasei* K5  
*L. paracasei* SP2  
*L. paracasei* SP5  
*Lactobacillus* AGR 4

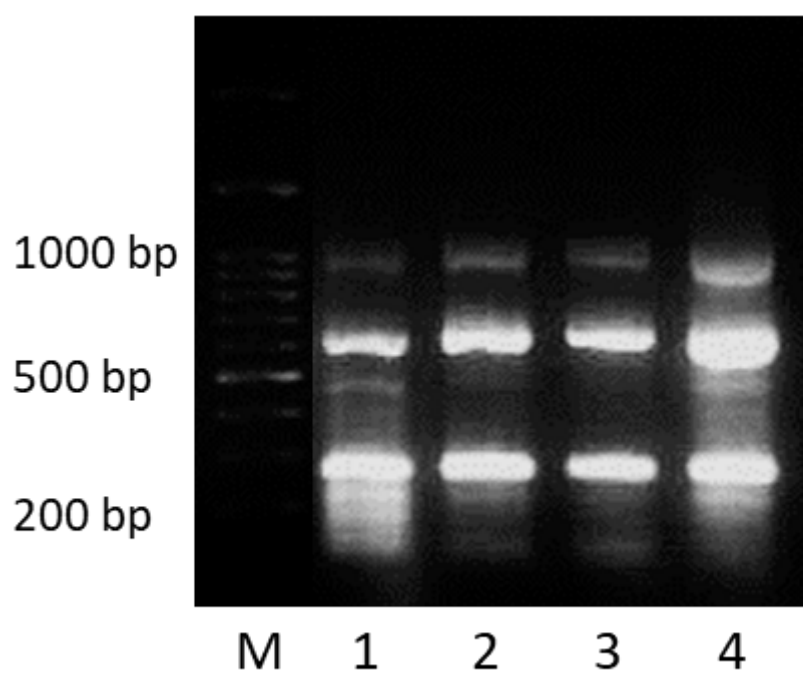

Supplement: Supplementary file 1 [file biomedicines-08-00594-s001.pdf]
